# Magnitude and predictors of first-line antiretroviral therapy regimen change among HIV infected adults: A retrospective cross sectional study

**DOI:** 10.1016/j.amsu.2022.104303

**Published:** 2022-08-17

**Authors:** Niguse Meles Alema, Solomon Weldegebreal Asgedom, Mahlet Maru, Beletu Berihun, Teklu Gebrehiwet, Tesfay Mehari Atey, Desalegn Getnet Demsie, Abere Tilahun Bantie, Adane yehualaw, Chernet Taferre, Sofia Assen Seid, Timsel Girma, Mengesha Dessie Allene, Sintayehu Mulugeta Tamru

**Affiliations:** a Department of Pharmacy, College of Medicine and Health Sciences, Adigrat University, Ethiopia; b Department of Clinical Pharmacy, School of Pharmacy, College of Health Sciences, Mekelle University, Ethiopia; c Department of Pharmacy, College of Medicine and Health Sciences, Bahir Dar University, Ethiopia; d Department of Anesthesiology, College of Medicine and Health Sciences, Dilla University, Ethiopia; e Department of Anesthesiology, College of Medicine and Health Sciences, Debrebirhan University, Ethiopia; f Department of Anesthesiology, College of Medicine and Health Sciences, Mekelle University, Ethiopia

**Keywords:** Prevalence, Reasons, HAART change, Adult HIV patients, Predictors, Ethiopia

## Abstract

**Background:**

Regimen change remains a significant challenge towards the achievement of human immunodeficiency virus (HIV) treatment success. In developing countries where limited treatment options are available, strategies are required to ensure the sustainability and durability of the starting regimens. Nevertheless, information regarding the rate and predictors of regimen change is limited in these settings.

**Objective:**

This study was undertaken to determine the prevalence and predictors of changes in ART regimens among patients initiating highly active antiretroviral therapy (HAART) at XX.

**Materials and methods:**

An institutional based retrospective cross-sectional study was conducted among adult naïve HIV patients who had initiated HAART at XX between 2010. Data were extracted by reviewing their medical charts using a pretested structured check-list. The Kaplan–Meier survival analyses were used to describe the probability of ARV regimen changes while Cox proportional hazard regression models were employed to identify the predictors of ARV regimen modifications. Data were analyzed using SPSS version 21 software, and statistical significant was deemed at p < 0.05.

**Results:**

A total of 770 patients were enrolled in this study of these 165 (21.43%) had their ART regimen modified at least once. Drug toxicity was the main reason for regimen change followed by TB comorbidity, and treatment failure. Positive baseline TB symptoms (aHR = 1.63, p = 0.037), and Zidovudine based regimen (aHR = 1.76, p = 0.011) as compared to Stavudine based regimen were at higher risk of ART modification. Conversely, urban residence, baseline World Health organization (WHO) stage 2 as compared to WHO stage 1, baseline CD4 count ≥301 as compared to CD4 count ≤200 were at lower risk of ART modification.

**Conclusion:**

The rate of initial HAART regimen change was found to be high. Thus, less toxic and better tolerated HIV treatment options should be available and used more frequently. Moreover, early detection and initiation of ART by the government is highly demanded to maximize the benefit and reduce risk of ART modifications.

## Highlights

• The majorities (57.4%) of the patients were female and the mean age of the study participants was 32.9 ± 9.5
• A total of 165 (21.4%) participants experienced antiretroviral regimen changes within two years of follow-up period.
• The highest rate of treatment modification was found among patients receiving fixed-dose combination of AZT+3 TC + NVP 81 (49.0%).
• Drug toxicity was the main reason for ARV regimen change, which accounts for 74 (41.3%) of regimen change.
• Anemia was the main forms of toxicities which accounted for 45(36.3%).

## 1 Introduction

Human immunodeficiency virus (HIV) is one of the major causes of morbidity and mortality, and continues to be a major public health concern worldwide [1]. It is thought that more than 32 million people were died, 1.7 million people became newly infected and 37.9 million people have been living with HIV in 2018 globally.

Sub-Saharan Africa remains the region that is suffering the most from HIV. A total of 20.6 million people were infected with HIV in Eastern and Southern Africa and 13.8 million people received antiretroviral therapy, which is 67% of HIV positive people in the region in 2018 [2]. Ethiopia is also one of the countries most affected by HIV infection and continues to pose a threat to people's lives. There were 786,040 HIV-infected [3] people in Ethiopia, 39,140 new HIV infections and 28,650 HIV/AIDS-related deaths in 2015 [3].

In June 2018, around two-thirds of HIV-positive people (23.3 million) receive life-saving ART worldwide [2]. The use of highly active antiretroviral therapy (HAART) in HIV care has dramatically decreased disease-related morbidity and mortality. It has also substantially increased the survival and quality of life of HIV patients and changed this pandemic from high mortality disease to manageable chronic diseases [1,4,5]. The main goal of ART is to achieve maximum and long-lasting suppression of viral replication which helps the immune system to recover and thus reduces the risk of opportunistic infections and death. However, treatment change and poor adherence limit the therapeutic success and durability of the original regimen. HAART regimens commonly require modifications, often involving switches of multiple medicines at the same time [6,7]. Early modification or change of initial HAART has been associated with poor clinical outcomes and a higher risk of treatment failure [8,9]. It also reduces the likelihood and duration of viral control due to cross-resistance and overlapping toxicity between and within the antiretroviral drug classes [4,10,11]. Optimizing the available restricted combination of antiretroviral regimen is, therefore, necessary to enhance the long-term success and longevity of the HIV treatment program.

Previous studies conducted in Ethiopia and other developing countries indicated that the original modification of the ART protocol was caused mainly by several reasons such as drug toxicities, inadequate adherence, development of treatment failure, presence of comorbidities, new drug availability, medication stockpiling, suboptimal protocol, and preference for pregnancy [[11], [12], [13], [14], [15], [16]].

In resource-constrained environments like Ethiopia, where treatment options are limited, developing strategies to increase the durability of the first regimen is fundamental. To achieve this goal, it is crucial to determine the magnitude, causes, and predictors of the first-line regimen change in the HAART. Knowledge of the main reasons and predictors of ARV regimen change could improve the success of initial HAART and minimize regimen change, treatment failure, drug resistance, and improve the patient's quality of life. However, data on the prevalence, causes, and predictors of ART regimen change have remained limited in the study area. This study was therefore carried out to determine the prevalence, reasons, and predictors of ARV regimen modification among patients initiating HAART.

## 2 Materials and Methods

### 2.1 Study design, area and period

An institution based cross-sectional retrospective study was conducted at ACSH from January 2021 to May 2021. ACSH is the largest comprehensive specialized hospital in the Tigrai region. It serves as a referral and teaching hospital and it provides health care services for patients referred from all general hospitals of the region and surrounding Amhara and Afar regional states. The hospital has more than 500 inpatient beds in four major departments and other specialty units and provides various health care services for a catchment population of approximately 10 million people. The hospital has an HIV/AIDS clinic that provide free clinical services, counseling and care to people living with HIV/AIDS starting from 2008. This research was retrospectively registered at www.researchregestry.com with Research Registry UIN of research registry number 7959 and reported according to STROCSS [17].

**Eligibility criteria:** All HIV adult patients (16 years or older), who had regular follow-up at the HIV clinic of the hospital from 2010 to 2020 and starting HAART.

**Exclusion criteria:** Patients with incomplete medical records, outpatient transfers, patient transfer to other institution, lost and dropped patients were excluded from the study.

### 2.2 Sample size determination

Single population proportion formula ($N=\frac{z^{2}P\left(1-P\right)}{d^{2}}$) was used to calculate sample size with the following assumptions. Where N is the needed sample size; d, marginal error (d = 0.05); Z, the required degree of accuracy at 95% confidence level, which is 1.96; p = 0.5 (50%) prevalence of regimen change, as there was no study conducted previously in this area to the best of literature search made using the above formula, the sample size was calculated as follows:

$$N=\frac{z^{2}P\left(1-P\right)}{d^{2}}\;=N=\frac{{\left(1.96\right)}^{2}0.5\left(1-0.5\right)}{0.05^{2}}=N=\frac{\left(3.8416\right)\left(0.25\right)}{0.05^{2}}=\;N=\frac{0.9604}{0.0025}=384$$

This is the minimum sample size in cross-sectional study and this study also retrospective chart review so chance of getting incomplete record is high so in order to increase appropriate representation the sample size was become double so final sample size was 770 patient medical records were reviewed.

### 2.3 Sampling technique

There were 2314 patient medical records in electronic media at Head of the HIV/AIDS clinic that had regular follow-up from 2010 to 2020 at ART clinic. A systematic random sampling technique was applied to select participants using the order of medical record number the first eligible participant chart was selected by lottery method and k is determined by dividing the all medical chart that had regular follow up from 2010 to 2020 to sample size which is 3($\frac{N}{n}\;=\frac{2314}{770}\;=3$) so every 3interval medical charts were reviewed to the study.

### 2.4 Study variables and measurements

#### 2.4.1 Independent variables

Socio-demographic variables such as age, sex, marital status, baseline weight, baseline BMI, place of residence, HAART regimen, WHO clinical stage, baseline CD4 count during HAART initiation, baseline functional status, baseline TB symptom, and year of HAART initiation were c,LNBHonsidered as independent variables of this study.

#### 2.4.2 Dependent variable

Prevalence of regimen change; time from initiation to first regimen change).

#### 2.4.3 Operational definition

**Regimen change**: Any alteration/switching or discontinuation of at least one antiretroviral drug from the triple ART regimen [19,20].

**Time of first regimen change:** first adjustment of the ARV regime recorded during the two years follow-up period of HAART's initiation.

**Immunologic failure**: According to the WHO guideline immunological failure was defined as a 50% decrease in the CD4^+^ count from the peak value of treatment, or a CD4^+^ count persistently below 100 cells/mm or drop of CD4^+^ count to pretreatment value (or below) [18].

**Clinical failure**: It was defined as the development of new opportunistic infection (WHO clinical stage 4 conditions) after six months of effective treatment.

**Functional status has been classified:** working (the ability to perform regular work in and out of the home), ambulatory (the ability to perform daily living activities) and bedridden (not capable of performing daily living activities).

### 2.5 Data collection tool and procedures

A structured data collection checklist, adapted from the ART National Standard Registry and through a review of the relevant literatures, was used to retrieve important information from patient medical records and charts about socio demographic, clinical data and antiretroviral treatment related information like baseline WHO stage, baseline CD4 count, date of initial regimen started, initial regimen, duration of initial ARV therapy before first switch made, regimen switch to, and reasons of regimen change.

Prior to the actual data collection, pre-testing was conducted on 39 HIV patients at Adigrat general hospital. Based on this pretest result necessary modifications were made on the data collection tool through in-depth discussion with responsible experienced internists and pharmacists working in HIV clinics of the hospital.

### 2.6 Data quality control

The data was collected by two trained pharmacists working outside the hospital and frequent checks were carried out on a daily basis by assigned supervisors in addition, the principal investigator carefully entered the data and cleaned it thoroughly before the data analysis began.

### 2.7 Statistical analysis

Data were analyzed using Statistical Package for Social Sciences (SPSS) program version 21. Descriptive statistics such as frequency and percentage distribution have been implemented to summarize socio-demographic, anthropometric and clinical features, as well as patterns and causes for changes in the ART regimen. The Kaplan–Meier product-limit method was used to plot survival curves. The probability of change in the ARV regimen and the median time to the endpoint was estimated using Kaplan Meier survival analysis. Time was measured from the beginning of HAART and ended at the earliest change/switch of regime, discontinuation, death, or 24 months follow up. Cox Proportional Hazard Regression Models with 95% confidence interval was used to estimate the hazard ratio of ART modifications and determine the factors associated with ARV regimen change. All tests of significance were two-sided and p-values of less than 0.05 were considered to be significant.

## 3 Results

### 3.1 Demographic and clinical characteristics of patients

A total of 770 HIV patients starting HAART chart have been reviewed. The majorities (57.4%) of the patients were female and the mean age of participants was 32.9 ± 9.5. Regarding their baseline functional status at the time of HAART initiation, 26.4, 63.2, and 10.4% were ambulatory, working and bedridden respectively (Table 1).

**Table 1:** Demographic and clinical characteristics of HIV patients at ACSH from 2010 to 2020.

Variable		N (%)
Age (in year)	15–29	294(38.2)
	30–44	390()50.6
	≥44	86(11.2)
Sex	Female	442(57.4)
	Male	328(42.6)
Residence	Rural	190(24.7)
	Urban	580(75.3)
Why eligible	Clinical only	45(5.8)
	CD4^+^ count	547(71)
	Others	178(23.2)
Baseline Functional Status	Ambulatory	203(26.4)
	Working	487(63.2)
	Bedridden	80(10.4)

Majority patients, 545 (70.8%) were started HAART based on CD4 count and most of them 603 (78.3%) were negative for baseline TB screening and around (72.7%) patients began treatment at WHO stage III and stage IV. There were six different drug combinations were prescribed as initial HAART regimens and Lamivudine (3 TC) was included in all combinations (Table 2).

### 3.2 Prevalence and predictors of ARV regimen change

As Cox's proportional hazards regression model showed that, Urban residence(aHR) = 0.45, 95%CI:0.29–0.68, p < 0.001], Baseline WHO stage 2 (aHR = 0.28, 95%CI:0.12–0.64, p = 0.002),Baseline CD4 count ≥301 (aHR = 0.39, 95%CI = 0.16–0.93, p = 0.034),TB symptoms (aHR = 1.63, 95%CI = 1.03–2.58, p = 0.037), and AZT+3 TC + NVP/EFV regimen (aHR = 1.757, 95%CI:1.14–2.71, p = 0.011) were found to be significant predictors for ARV regimen change (Table 3) (see Table 4).

**Table 2:** Overall patterns of the HIV/AIDS patients on HAART at ACSH from 2010 to 2020.

Characteristics	N (%)
**Baseline WHO stage**	
T1	113(14.7)
T2	97(12.6)
T3	410(53.2)
T4	150(19.5)
**Baseline TB screen**	
Positive	167(21.7)
Negative	603(78.3)
**Baseline ARV regimen**	
TDF+3 TC + EFV	312(40.5)
AZT+3 TC + NVP	235(30.5)
d4T+3 TC + NVP	100(13)
TDF+3 TC + NVP	79(10.3)
d4T+3 TC + EFV	26(3.4)
AZT+3 TC + EFV	18(2.3)

**Notes:** WHO: World Health Organization; TB: tuberculosis; CD4+: cluster of differentiation 4; ARV: antiretroviral; TDF: Tenofovir; 3 TC: lamivudine; EFV: efavirenz; NVP: nevirapine; AZT: zidovudine; D4T: stavudine.

**Table 3:** Predictors for first line ART modification among HIV patients at ACSH from 2010 to 2020.

Variables	Regimen change			aHR (95%CI), *p*-value
	No (%)	Yes (%)		
Residence				
Rural	146(24.1)		44(26.7)	1
Urban	459(75.9)		121(73.3)	0.45(0.29–0.68), p = 0.000*
Baseline functional status				
Ambulatory	156(25.8)		47(28.5)	1
Working	390(64.5)		97(58.8)	0.75(0.47–1.21), p = 0.25
Bedridden	59(9.8)		21(12.7)	1.17(0.58–2.34), p = 0.657
Baseline WHO stage				
T1	92(15.2)		21(12.7)	1
T2	86(14.2)		11(6.7)	0.28(0.12–0.63), p = 0.002*
T3	311(51.4)		99(60)	0.60(0.357–1.01), p = 0.053
T4	116(19.2)		34(20.6)	0.74(0.33–1.66), p = 0.473
Baseline TB symptoms				
Positive	125(20.7)		42(25.5)	1.63(1.03–2.58), p = 0.037*
Negative	480(79.3)		123(74.5)	1
Baseline CD4 count				
≤200	426(70.4)		139(84.2)	1
201-300	112(18.5)		15(9.1)	0.60(0.32–1.13), p = 0.114
≥301	67(11.1)		11(6.7)	0.39(0.16–0.93), p = 0.034*
ARV regimen				
d4T+3 TC + NVP/EFV	73(12.1)		53(32.1)	1
TDF+3 TC + EFV/NVP	366(60.5)		25(15.2)	0.75(0.43–1.29), p = 0.33
AZT+3 TC + NVP/EFV	166(27.4)		87(52.7)	1.75(1.14–2.71), p = 0.011*

**Notes:** 1 Reference category,*statistically significant at *p* < 0.05.

**Abbreviations:** aHR: adjusted hazard ratio; CI: confidence interval.

The log-rank test was used to estimate the time difference between groups to change the ARV regimen in months. Hazard curves plots of the place of residence [Log Rank (Mantel-Cox) (p = 0.002)], baseline functional status [Log Rank (Mantel-Cox) (p = 0.003)], baseline WHO stage [Log Rank (Mantel-Cox) (p = 0.001)], baseline CD4 count [Log Rank (Mantel-Cox) (p = 0.009)], ART regimen [Log Rank (Mantel-Cox) (p < 0.001)] and baseline TB screening symptoms [Log Rank (Mantel-Cox) (p < 0.001)] were statistically significantly influenced the time to change ARV regimen (**Additional file 1–6**).

### 3.3 Antiretroviral treatment modifications/changes

**Fig. 1:**
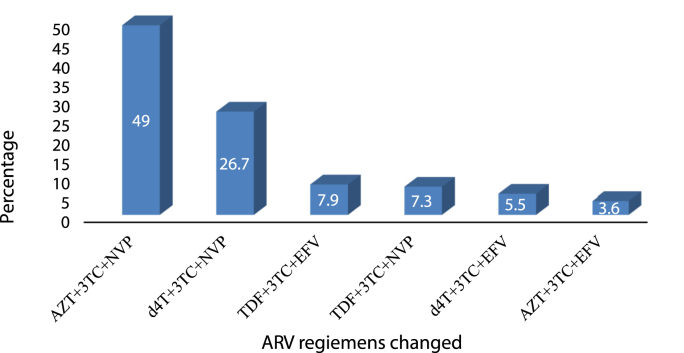
Percentage distribution of frequently changed ARV regimen of HIV patients at ACSH from 2010 to 2020.

From 770 participants starting ARV medication around 165 (21.4%) participants experienced antiretroviral regimen changes during their follow-up period. The highest rate of treatment modification was found among patients receiving fixed-dose combination of AZT+3 TC + NVP followed by D4T+3 TC + NVP and TDF+3 TC + EFV which represents approximately 81 (49.0%), 26.7% (44), and 7.9% [13] of regimen changes consecutively (Fig. 1).

**Fig. 2:**
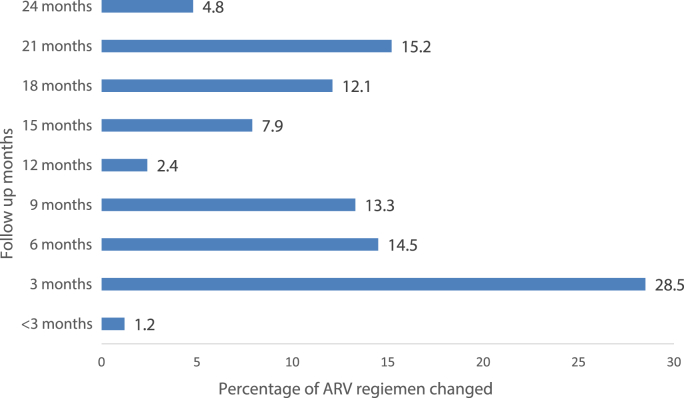
Percentage distribution of ARV regimen modified with respect to the follow-up months among HIV patients in at ACSH from 2010 to 2020.

The median of time to change the first ARV regimen in months was found to be 9 months and the average mean time to change the ARV regimen was 10.93 ± 7.4 months. As indicated in Fig. 2 below, among the total 165 patients who changed their first ARV regimen in the current study, a significant proportion 47 (28.5%) of the patients changed their regimen in the first three months of follow-up and very few 2 (1.2 per cent) patients changed their ARV regimen in less than 3 months of follow-up.

**Fig. 3:**
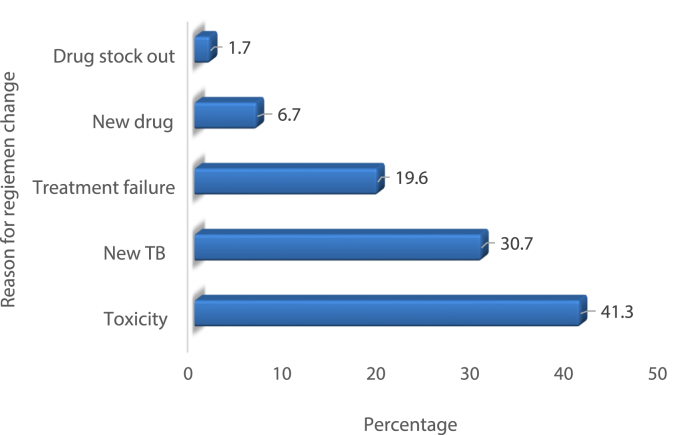
Reasons of ART modification among HIV patients who changed their first line ART.

As shown in Fig. 3, the main reason for ARV regimen change was drug toxicity, which accounts for 74 (41.3%) of regimen change, followed by new TB development (30%), and treatment failure (19.6%). As indicated Table 4 anemia was reported as main forms of toxicities which accounted for 45(36.3%) followed by rash 23 (18.5%), peripheral neuropathy 21(17%), lip atrophy 13(10.5%), and central nervous system toxicity 8(6.5%). The majority of toxicities were caused by Zidovudine (AZT), Stavudine (D4T), and Nevirapine (NVP) containing regimens. Peripheral neuropathy and lipoatrophy was caused by stavudine containing regimens of D4T+3 TC + NVP/EFV, while anemia was reported due to Zidovudine containing regimens of AZT+3 TC + NVP/EFV. Rash was reported mainly due to Nevirapine containing D4T+3 TC + NVP, AZT+3 TC + NVP and TDF+3 TC + NVP regimens. In addition, central nervous system toxicity such as dizziness, nightmares, and sleep disturbances was reported due to efavirenz (EFV)-containing regimens.

**Table 4:** Drugs and types of toxicities reported as reasons for initial regimen switch in ART clinic, at ACSH from 2010 to 2020.

Start regimen	anemia	rash	peripheral neuropathy	Lip atrophy	central nervous system toxicity	Total
TDF+3 TC + EFV	0(0%)	0(0%)	0(0%)	0(0%)	3(100%)	3(100%)
AZT+3 TC + NVP	38(73%)	14(27)	0(0%)	0(0%)	0(0%)	52(100%)
D4T+3 TC + NVP	0(0%)	7(33.33)	14(66.67%)	0 (0%)	0(0%)	21(100%)
TDF+3 TC + NVP	0(0%)	2(20%)	0(0%)	8(80%)	0(0%)	10(100%)
D4T+3 TC + EFV	0(0%)	0(0%)	7(50%)	5(35.71%)	2(14.23%)	14(100%)
AZT+3 TC + EFV	7(70%)	0(0%)	0(0%)	0(0%)	3(30%)	10(100%)
Total	45(36.3%)	23 (18.5%)	21(17%)	13(10.5%)	8(6.5%)	110(100%)

## 4 Discussion

The change to the ART protocol affects the effectiveness of antiretroviral drug therapy and limits the need to meet the objectives of the United Nations HIV and AIDS Program [18]. Our research provides important insights into the causes of HAART regimen modification in HIV/AIDS patients on HAART regimen and describes the rate of change in the HAART regimen, the common reasons for changes in the regimen, and the predictors of modifications in the ARV regimen.

In the current study, the prevalence of ARV regimen change was found to be 21.4%. This finding was consistent with previous studies conducted at referral hospital in Ethiopia, which reported prevalence of ARV regimen change of 21.5% and 20.3% in Gondar Hospital University [19,20]. On the contrary, it is much lower than the studies conducted somewhere else in Ethiopia which reported a high rate of ARV regimen changes ranging from 33.9% to 62.8% [13,21,22]. Higher rates of regimen change have been reported in cohort studies in developed countries such as Brazil (69%), Italy (36%), France (68%), and Europe and North America [[23], [24], [25]]. The high rate of ART modification could be due to difference in defining the regimen change, and the availability of a relatively higher number of HAART treatment options in resource rich countries compared to developing countries like Ethiopia [11]. Moreover, the recent availability of ultrasensitive viral load tests that favor early detection of active viral replication and thus virological failure, which could potentially increase the rate of ARV regimen changes in resource-rich countries [11].

Our study revealed that drug toxicities (41.3%), TB infection (30.7%), and treatment failure (19.6%) were the top three causes for the change of the first-line regimen. This result is consistent with previous studies conducted in developing and developed countries [16,[23], [24], [25], [26], [27]]. Drug toxicity was the primary cause of the ART regimen change. This is mainly caused due to the inherent properties of the drugs which are aggravated by late initiation of HAART [28,29]. Similar to other studies, anemia was the predominant reason for regimen change among the numerous toxicities identified in this study [14,30]. However, this was not in line with other studies conducted in some parts of Ethiopia and Kenya, which reported peripheral neuropathy as the main reason for ART modifications [12,26,31]. This difference could be explained by the fact that most of the patients enrolled in our study initiated the AZT-based regimen.

The second main reason responsible for the ART regimen switch was the development of new TB. Co-infection with TB and HIV leads to a drug interaction between HAART and anti-TB drugs. TB medications, specifically rifampicin, induce *Cytochrome* P450 *3A4* that facilitates the metabolic activity of the liver, which ultimately reduces the therapeutic level of ART drugs, particularly nevirapine. Some studies also demonstrated the overlapping toxicities when the drugs are administered together [[32], [33], [34]]. The drug-drug interaction between nevirapine and rifampicin were responsible for the TB-related switches.

The hazard of changing the HAART regimen for patients who started AZT+3 TC + NVP/EFV regimen was 1.76 times higher than of these patients who started d4T+3 TC + NVP/EFV regimen. This may be explained by the frequent use of AZT based fixed-dose combination that is associated with adverse effects like anemia. In this study, a higher record of hematological toxicity (36.3%) was found. This is in concordance with other studies [28]. Hence, patients with anemia at baseline should be typically assigned to non-AZT based regimen.

The hazard of regimen changes on patients who had TB positive symptoms at baseline was 1.63 times higher than patients having negative TB symptoms. This is consistent with the findings of previous studies [13,35,36]. The plausible justification is that those patients with positive TB symptoms might develop TB infection. Then drug interaction between the ant-TB medications and ART drugs might result in modification of the regimen.

Patients from urban residences, patients with baseline WHO stage II, and patients who had a baseline CD4^+^ count of ≥301 cells/mm^3^ had a lower hazard for regimen change (Table 3). Patients from urban residences might have better awareness and knowledge and were also proximate to follow-up areas. Hence could have less complained of the adverse effects which might decrease the ARV regimen modifications compared to patients who live in rural areas. Moreover, their good knowledge might insist on good adherence, thus could enhance ARV therapy effectiveness, which could minimize treatment failure and opportunistic infection.

Patients with baseline CD4^+^ lymphocyte count of ≥301 cells/mm^3^ were less likely to change regimen than those with a CD4^+^ lymphocyte count of ≤200 cells/mm^3^. A higher baseline CD4^+^ lymphocyte count was closely related to the slower progression of HIV disease [37]. Unfavorable trends in CD4^+^ lymphocyte counts are an indication of drug failure and suggest a change in the HAART regimen or intensification of opportunistic infection prophylaxis before new clinical events [38]. Thus, higher baseline CD4^+^ counts may decrease disease progression and thus reduce ARV regimen changes.

### 4.1 Implication for practice

Antiretroviral therapy (ART) has markedly decreased the morbidity and mortality due to HIV/AID. But frequent change of antiretroviral Treatment (ART) regimen is a challenging problem especially in a resource-limited setting like Ethiopia where treatment options are limited. This study was aimed to identify reasons for ART regimen change among adult HIV patients and mitigating strategy is required by different stakeholders to maintain patients with HIV on their initial regimen.

### 4.2 The implication for further research

A body of evidence revealed that antiretroviral treatment (ART) regimen change is high. Therefore, ART utilization and factors associated with modification of HAART should be investigated further in prospective longitudinal studies. Selection of the right antiretroviral regimen and appropriate care, close follow-up and frequent laboratory result monitoring after ART initiation is necessary to maintain patients on their initial regimen.

### 4.3 Limitations

Our study is not without flaws. One of the limitations of our study was incomplete information in the medical records of the patients. Some of the important demographic and clinical variables, such as income and viral load, were not appropriately registered in the patients chart. Thus the treatment failure we collected from medical records of the patients were immunologic/or clinical failure rather than virologic failure which is the confirmatory test of treatment failure. Another limitation to our study was due to the absence of a standardized schedule of laboratory tests, laboratory values like baseline AST, ALT and hemoglobin levels were poorly recorded in the patients’ medical chart and thus were excluded from analysis.

## 5 Conclusion and recommendation

We found higher prevalence of ARV regimen change mainly caused due to drug toxicity, new TB development, and treatment failure. Urban residence, baseline WHO stage 2, baseline CD4 count ≥301, positive baseline TB symptoms and AZT+3 TC + NVP/EFV regimen were strongly associated with ARV regimen change. Further follow-up using a multi-centered longitudinal cohort studies should be conducted to determine the long term clinical outcome of patients treated with HAART.

## Consent to publish

Not applicable.

## Availability of data and materials

The datasets supporting the conclusions of the study are included in the article and supplementary material. If any additional data is required it will be available based on reasonable request.

## Funding

The study was not supported by any funding agent.

## Provenance and peer review

No funding was obtained from any organization.

Not commissioned, externally peer-reviewed.

## Author contributions

All authors made a significant contribution to the work reported, whether that is in the conceptualization, data curation, formal analysis, investigation, methodology, supervision, validation, visualization; took part in writing - original draft, writing - review and editing; gave final approval of the version to be published; have agreed on the journal to which the article has been submitted; and agree to be accountable for all aspects of the work.

## Authors’ contributions

NMA, SWA, MM, BB, TG and TMA involved in the conception, reviewing, analysis, study design and drafted the manuscript. DGD, ATB, AY, CT, SAS, TG and MDA critically reviewed the manuscript for important intellectual content. All authors participated in the study design, supervised the development of the manuscript and involved in manuscript writing and editing. All authors read and approved the final manuscript.

## Ethical approval

Ethical clearance and approval was obtained from the ethical review committee Mekelle University, Health Science College. In addition, permission was sought from ACSH medical director and the Head of the HIV/AIDS clinic to obtain the patients' medical records. For the sake of maintaining patient confidentiality, personal identifiers like the name of patient were not recorded in the data collection tool.

## Guarantor

All authors.

## List of abbreviations

3 TC: Lamivudine; ACSH: Ayder Comprehensive Specialized Hospital; aHR: Adjusted Hazard Ratio; ALT: Alanine Lactate Transferase; ARV: Antiretroviral; AST: Amine Sulfate Transferase; AZT: Zidovudine; BMI: Body Mass Index; CI: Confidence Interval; D4T: Stavudine; EFV: Efavirenz; HAART: Highly Active Antiretroviral Therapy; HIV: Human Immune Virus; NVP: Nevirapine; TB: Tuberculosis; ART: Antiretroviral Therapy.
