# Supplementary material for: Magnitude and predictors of first-line antiretroviral therapy regimen change among HIV infected adults: A retrospective cross sectional study
Source: Ann Med Surg (Lond). 2022 Aug 17;81:104303. doi: 10.1016/j.amsu.2022.104303 (PMC9486446; doi:10.1016/j.amsu.2022.104303)
Supplement: Multimedia component 1 [file mmc1.docx]

**Additional figures**

# Additional file 1


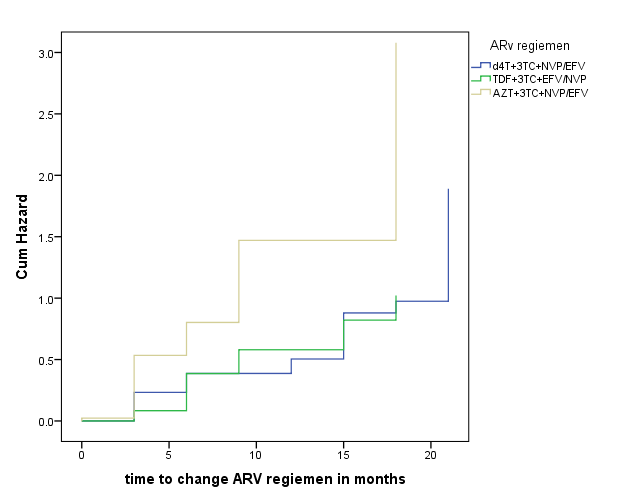


Figure1: Kaplan-Meier hazard curve for antiretroviral regimen among HIV patients by time to change antiretroviral regimen at HIV clinic of ACSH, between 2010 and 2020.


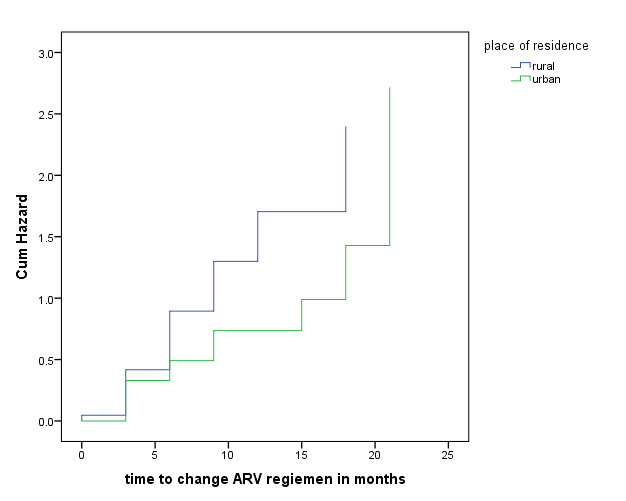


Figure 2: Kaplan-Meier hazard curve for place of residence among HIV patients by time to change antiretroviral regimen at HIV clinic of ACSH, between 2010 and 2020.


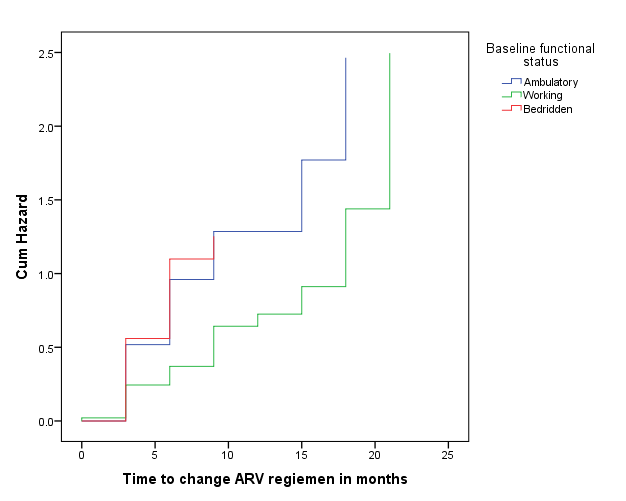


Figure 3: Kaplan-Meier hazard curve for baseline functional status among HIV patients by time to change antiretroviral regimen at HIV clinic of ACSH, between 2010 and 2020.


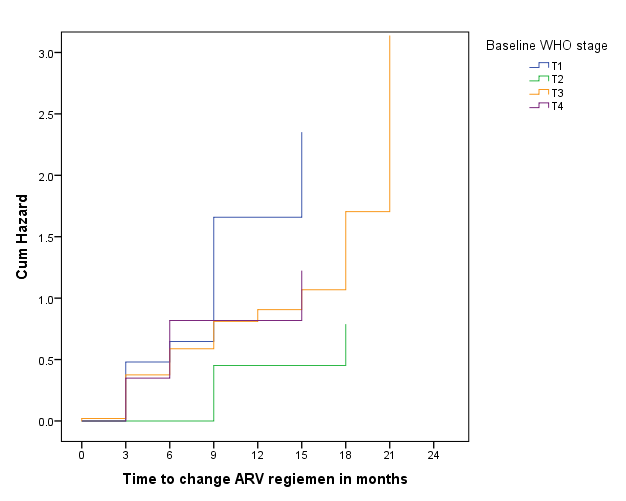


Figure 4: Kaplan-Meier hazard curve for baseline TB symptoms among HIV patients by time to change antiretroviral regimen at HIV clinic of ACSH, between 2010 and 2020.


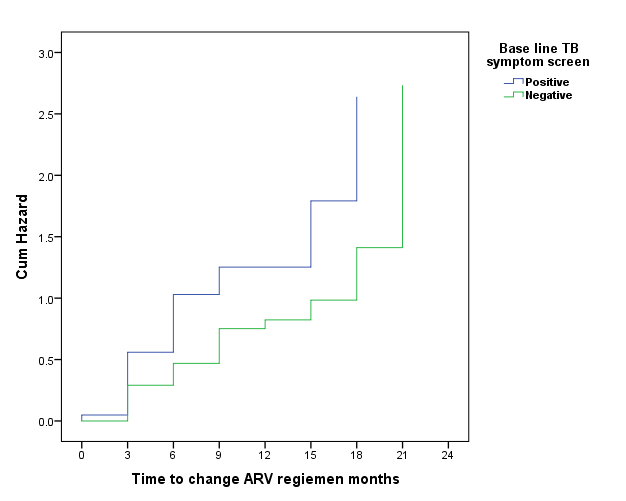


Figure 5: Kaplan-Meier hazard curve for baseline TB symptoms among HIV patients by time to change antiretroviral regimen at HIV clinic of ACSH, between 2010 and 2020.


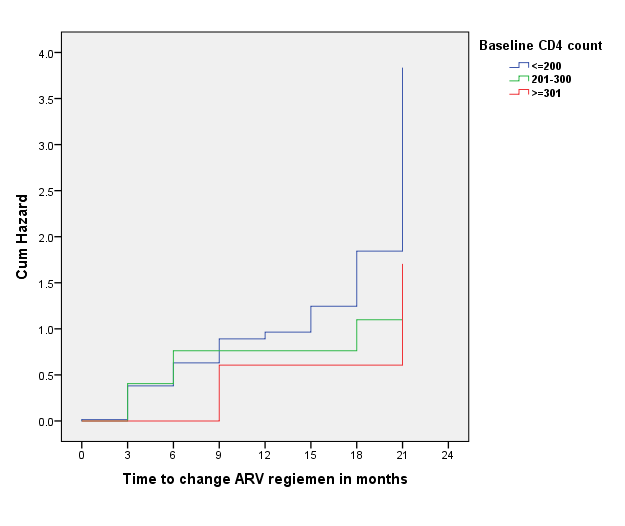


Figure 6: Kaplan-Meier hazard curve for baseline CD4 count among HIV patients by time to change antiretroviral regimen at HIV clinic of ACSH, between 2010 and 2020.
